# Case of Poisoning by Fluid Extract of Gelseminum

**Published:** 1868-12-01

**Authors:** W. F. Hani

**Affiliations:** Middlebury, Elkhart Co., Indiana


### CASE OF POISONING BY FLUID EXTRACT OF GELSEMINUM.

BY W. F. HANI, OF MIDDLEBURY, ELKHART CO., INDIANA.

PROF. ALLEN — *Kind Sir:*

As one of the Alumni of Rush Medical College, I would ask to transmit to the profession, through the columns of your JOURNAL, the report of a case of poisoning and death by the article in *Materia Medica* known as Gelseminum Sempervirens, or yellow jessamine.

J. F. came to my office on the evening of March 24th, 1868, and stated to me that his son F., aged eighteen months, had taken part of the contents of a vial of medicine which was given him to play with by his mother, and which had been prescribed for him about a month previously while laboring under an attack of acute pneumonia. After taking it he, in fifteen or twenty minutes, became so stupid that he could not be aroused by his mother. I then asked him how long since he had taken the dose, when he replied, about one-half hour since; upon which he handed me the vial that contained the fatal draught. Upon examination I found it contained the *Fluid extract of gelseminum.* I then told him that F., as I thought, had taken a poisonous dose of the article. He then wanted to know where Dr. J. Yagerlenner was, who had prescribed the medicine. I told him the doctor had just gone to his home, and he had better see the doctor immediately and have him see the boy. When the doctor arrived, he found the child retching and vomiting, and difficult to arouse. Dr. J. immediately gave him brandy, but only succeeded in getting him to swallow about a teaspoonful, when F. fell into profound stupor which lasted until life was extinct. About two hours subsequent to Dr. J.'s arrival I was sent for, and upon arriving and hearing the doctor's statement, I examined the case and found F. apparently lifeless, face and lips blue and livid, no pulse at the wrist nor at the carotids, pupils large and dilated, and upon examining the heart found the palpitation slow and very feeble, but continued to beat twenty minutes when life ceased. The amount of fluid extract taken by F., was, as stated by one of the parents, about thirty drops.

Now, as I am unable to find any authenticated cases of poisoning and death from the effects of yellow jessamine, I would be pleased to hear from some professional brother, through the columns of your JOURNAL, of any of the foregoing cases, and upon the best means and remedies adapted to counteract the poisonous effects of the yellow jessamine. My own opinion is inclined to be that emetics administered immediately with the stomach pump, and *Opium* given as an antidote, or some of the preparations of *Strychnia.* My reasons for resorting to *Opium* and *Strychnia* are based upon the fact that they produce contraction of the pupil — the direct opposite of the condition of the pupil in this case.

